# The NLRP3 Inflammasome: Relevance in Solid Organ Transplantation

**DOI:** 10.3390/ijms221910721

**Published:** 2021-10-03

**Authors:** Ryan M. Burke, Bethany L. Dale, Shamik Dholakia

**Affiliations:** 1 CareDx, Inc., Brisbane, CA 94080, USA; rburke@caredx.com (R.M.B.); bdale@caredx.com (B.L.D.); 2 Oxford Transplant Center, Nuffield Department of Surgical Sciences, University of Oxford, Oxford OX3 7LD, UK

**Keywords:** NLRP3, inflammasome, autoimmunity, alloimmune injury, donor-derived cell-free DNA, neutrophil extracellular trap, fibrosis, solid organ transplant

## Abstract

The NOD, LRR, and pyrin domain-containing 3 (NLRP3) protein has been established as a central component of the inflammasome and regulates the inflammatory response to a myriad of environmental, microbial, and endogenous danger stimuli. Assembly of the NLRP3 inflammasome results in the cleavage and activation of caspase-1, in turn causing release of the pro-inflammatory interleukins 1-beta and 18. This activation response, while crucial to coordinated innate immune defense, can be aberrantly activated by the likes of cell-free DNA, and cause significant autoimmune pathology. Complications of autoimmunity induced by aberrant NLRP3 inflammasome activation have a great degree of mechanistic crossover with alloimmune injury in solid organ transplant, and stratagems to neutralize NLRP3 inflammasome activation may prove beneficial in solid organ transplant management. This article reviews NLRP3 inflammasome biology and the pathology associated with its hyperactivation, as well as the connections between NLRP3 inflammasome activation and allograft homeostasis.

## 1. Composition and Assembly of the NLRP3 Inflammasome

NLRP3 is a three-domain protein containing an N-terminal pyrin domain, a central nucleotide-binding and oligomerization domain (NOD), and a C-terminal leucine-rich repeat domain [1,2]. Inflammasome assembly is initiated when the pyrin domain of NLRP3 interacts with the pyrin domain of the apoptosis-associated speck-like protein containing a CARD (ASC), an interaction that has been extensively analyzed and fully structurally solved by NMR [3,4]. Upon NLRP3 interaction with ASC via the pyrin domains, ASC fibrils self-assemble into ‘speck’ structures with the primary function of pro-caspase-1 recruitment and the subsequent auto-activation of caspase-1 by proteolysis—representing the fully assembled NLRP3 inflammasome [5,6].

**Figure 1:**
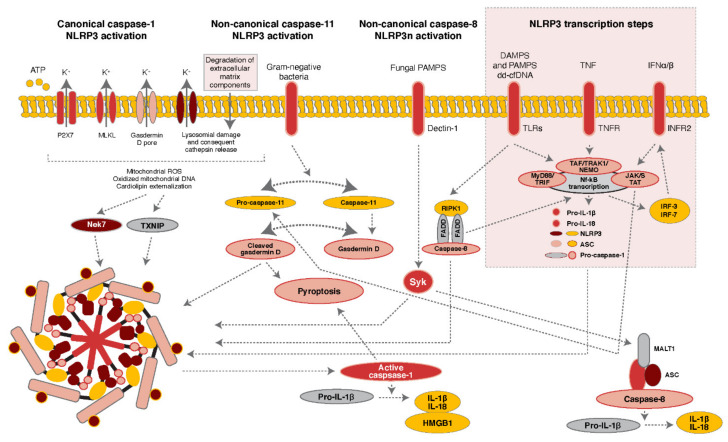
NLRP3 inflammasome signaling in transplantation. This diagram displays the molecular mechanisms underlying NLRP3 inflammasome activation, with an emphasis on those specific to solid organ transplant. *Classical Canonical NLRP3/caspase-1 activation:* P2X7 receptor activation by extracellular ATP leads to MLKL activation. Exposure to pore-forming gasdermin D combined with lysosomal damage and cathepsin release promote potassium efflux, with consequent release of oxidized mitochondrial DNA and dd-cfDNA—leading to increase in mitochondrial ROS and cardiolipin externalization. This in turn promotes NLRP3 inflammasome oligomerization and activation with the consequent release of IL-1β,IL-18, and HMGB1 and the autocatalytic cleavage of caspase-1. This active form of caspase-1 is also a major driver of the cell death pathway pyroptosis (see main text). *Non-canonical caspase-11-dependent NLRP3 activation—bacterial infection in transplant:* Gram-negative bacteria activate caspase-11, cleaving pore-forming gasdermin D between the N-terminal and C-terminal domains. Cleaved gasdermin D promotes pyroptosis and the activation of canonical NLRP3-ASC-caspase-1 signaling. *Non-canonical caspase-8 NLRP3 activation—fungal infection in transplant:* TLRs activation by PAMPs and/or DAMPs such as dd-cfDNA activate the RIP1–FADD-caspase-8 pathway and NF-kB alongside the promotion of canonical NLRP3 activation. Moreover, fungal PAMPS, via dectin-1 activation, promote the activation of the MALT1–caspase-8–ASC complex, which promotes the processing and release of IL-1β. *NLRP3 transcription steps:* Transcription steps are regulated by TLRs—MyD88, TNFR and/or IFNα, β-JAK/STAT, which, promoting NF-κB activation, induce the transcription of pro-IL-1β, NLRP3, procaspase-1, IRF-3 and IRF-7, and procaspase-11. The activation of TLRs stimulates the formation of RIP1–FADD-caspase-8 complex accelerating NF-kB transcription.

Regulation and activation of NLRP3 has been established to require two sequential signals—a priming signal and an activation signal [7,8,9,10]. Priming is accomplished by agonists of the pattern recognition receptor (PRR) family of Toll-like receptors (TLRs), which are primarily expressed on the cell surface or endosomes of innate immune cells such as dendritic cells (DCs) and macrophages, as well as other non-immune cell types such as fibroblasts and epithelial cells [11,12]. TLRs are largely classified into two subfamilies based on their localization, cell surface TLRs and intracellular TLRs. The main functions of TLRs are to recognize both damage-associated molecular patterns (DAMPs) that derive from endogenous cells under stress or undergoing death processes as well as pathogen-associated molecular patterns (PAMPs) that are characteristic of microbial invaders. The canonical example of TLR4 and its ability to recognize bacterial lipopolysaccharide (LPS) is often used to illustrate the NLRP3 assembly and activation response to PAMPs [13]. The specialized recognition functions, specific roles in the immune response, and regulation/pharmacological targeting strategies of the 10 known human TLR proteins are beyond the scope of this article, but are reviewed in excellent detail elsewhere [14,15,16,17]. Of interest to this article are the subset of TLRs which recognize extracellular DNA debris. TLR sensing of DAMPs and PAMPs inevitably results in downstream signaling that leads to the nuclear translocation of the transcription factor NF-κB, which directly regulates (amongst many other things) the expression of NLRP3 and pro-IL-1β, neither of which is expressed in resting macrophages at significant levels [18,19]. Other elements involved in regulating the expression of these two molecules (mainly NF-kB pathway and apoptotic signaling components) also have been shown to specifically regulate NLRP3 and pro-IL-1β expression, and are comprehensively reviewed elsewhere [1]. Non-transcription-dependent priming signals have also been discovered, and these are discussed in greater detail in Section 2.1. The activation signal follows the priming signal, and is transduced by numerous mechanisms including alterations in ionic flux, mitochondrial DNA release, reactive oxygen species, and lysosomal damage and dysfunction. These pathways are elegantly reviewed in [1], and of particular interest to the field of organ transplant are the connections between DNA sensing and NLRP3 inflammasome activity. We also summarize and illustrate these pathways in Figure 1, and these connections are explored further in Section 3.

## 2. Regulation of NLRP3 Inflammasome Activity

### 2.1. Role of Post-Translational Modifications in NLRP3 Inflammasome Activation

#### 2.1.1. Phosphorylation of NLRP3 and Kinases Associated with Inflammasome Activation

NLRP3 inflammasome priming and activation are governed by multiple levels of phosphorylation-based oversight. Phospho-proteomic analysis by Flag-tag affinity purification paired to LCMS also identified NLRP3-S198 as a JNK1 target that, when phosphorylated during the priming process, promotes the formation of NLRP3 multimers that facilitate rapid inflammasome assembly [20]. The PP2A-dependent dephosphorylation of NLRP3-S5, which alleviates electrostatic repulsion that prevents runaway self-assembly of NLRP3 inflammasomes, has been shown to be indispensable in the initial phase of activation, known as ‘licensing’ [21]. Phosphorylation of NLRP3-S295 by PKA or PKD negatively regulates inflammasome activation, and S295A mutation recapitulates cryopyrin-associated periodic syndrome (CAPS) phenotypes [22]. The classically observed CAPS-associated clinical presentations (familial cold autoinflammatory syndrome, Muckle-Wells syndrome, and neonatal-onset multisystem inflammatory disorder) have been traced to heterozygosity for gain-of-function mutations in NLRP3 and are characterized by persistent inflammation ending in organ damage [23]. Similarly, PTPN22 was shown to mediate dephosphorylation of NLRP3-Y861 to negatively regulate inflammasome activation, and loss of function variants of PTPN22 have been implicated in various autoimmune diseases such as systemic lupus erythematosis, Crohn’s disease, rheumatoid arthritis, and type I diabetes [24].

Another chemical screening study demonstrated that inhibitors that target IKK or its downstream pathway are ineffective at limiting LPS/ATP-induced caspase-1 activation in vitro, but upstream inhibitors of the IκB kinase (IKK) complex effectively limit both NF-κB and NLRP3 inflammasome activation [25]. This study implicated TGF-β-activated kinase 1 (TAK1) as a potentially strong candidate for phosphorylation-dependent activation of NLRP3 inflammasome components and a likely mediator of the priming signal. TAK1 has been shown to regulate the myocardial response in a mouse model of pressure overload, by promoting adaptive cardiac hypertrophy via cross-talk between the NF-κB/IKK and calcineurin-NFAT pathways [26]. In this specific setting, TAK1 provides protection by countering anti-inflammatory fibrotic signals—and does so by promoting both pro-survival and inflammatory signaling milieus that in turn positively regulate cardiomyocyte survival. However, TAK1 signaling has also been associated a more nuanced effect in a rat model of myocardial infarction—it was found that NLRP3 inflammasome hyperactivation was associated with the death of cardiomyocytes by gasdermin D (GSDMD)-dependent pyroptosis and that TAK1/JNK1 signaling was an effective promoter of this hyperactivation [27]. Inhibition of TAK1/JNK1 signaling by the molecule LCZ696 (combined neprilysin inhibitor and angiotensin-II receptor blocker) inhibited pyroptosis-mediated cardiomyocyte death, and conversely constitutive overexpression of TAK1 specifically induced expression and caspase-1-dependent cleavage of GSDMD that reversed the effects of LCZ696. The authors concluded that targeted inhibition of TAK1 and JNK1 as central regulators of NLRP3 inflammasome activation and spontaneous cell death might provide a novel avenue for investigation of NLRP3 inflammasome pharmacologic inhibition.

Conversely, TAK1 silencing in macrophages has been shown to induce the spontaneous activation of the NLRP3 inflammasome without TLR priming and subsequent activation signals via uncontrolled secretion of TNF-α and abnormal signaling transmission [28]. The authors reported a heretofore unknown role for macrophage TAK1 in suppressing hyperactivation of IKKα/β that leads to subsequent over-release of TNF-α and NLRP3 inflammasome activation without priming or activation signal requirement. The authors followed these observations with an elegant study elucidating a novel kinase activity–independent role for RIPK1 in death processes using a model of TLR priming in a TAK1-deficient setting that recapitulated pathogen-induced priming and inhibition [29]. TLR priming of TAK1-KO macrophages was characterized by NLRP3 inflammasome assembly and subsequent recruitment of NLRP3 and ASC into a RIPK1 kinase activity–independent cell death complex. The authors build a compelling case that RIPK1 can serve a completely independent role in inflammatory cell death, and merits attention as a potential upstream signaling mediator of NLRP3 inflammasome activation in its own right.

The MAPK signaling cascade also is activated by at least two downstream adaptors in TLR signaling that have been shown to have direct roles in regulating NLRP3 and IL-1β expression levels [30,31]. p38 is a MAPK recently implicated in NLRP3 priming and reviewed in [32]. Importantly, isoforms of p38 were reported to be required for inflammasome activation in response to UV radiation, and the p38-MK2 signaling axis was specifically implicated at the priming signal step. Recently, the peptidyl-prolyl cis/trans isomerase Pin1 was implicated in regulating NLRP3 inflammasome component expression levels and level of activation in a p38-dependent manner, though Pin1 and p38 did not directly interact [33]. Additionally, a recent study in adipose tissue insulin resistance in response to statin therapy has implicated dysregulation in protein prenylation via statin activation of p38 upstream of IL-1β, which led to priming of NLRP3 inflammasomes in macrophages, even in mice lacking IL-1β, and promoted subsequent lack of prenylation and increased insulin resistance [34]. In the same vein as TAK1, extensive evidence for p38-mediated signal transduction in pathologic cardiac remodeling has been accumulated over the past decade, and illuminates some of the potential relevant cross-talk in signaling pathways in non-immune cell types. Cardiac fibroblasts give rise to cardiac myofibroblasts during pathologic cardiac remodeling largely through the effects of TGF-β1, resulting in increased fibrotic synthesis and altered genetic profiles that include an increase in expression of both TLR4 and NLRP3 [35]. Indeed, in this study, the authors discovered that the increase in TLR4 and NLRP3 expression in cardiac myofibroblasts was directly due to TGF-β1, and was transduced by p38 signaling—providing additional corroboration to the potential role for p38 signaling in NLRP3 inflammasome activation not just in immune cells, but in cell types that are less traditionally considered in terms of their contribution to systemic inflammatory signaling.

Extracellular signal-related kinases (ERKs) have also been recently implicated in NLRP3 inflammasome activation. One group reports LPS-induced phosphorylation of extracellular signal-regulated kinase (ERK) in immortalized macrophages in vitro, and showed that a biological extract of the filamentous cyanobacterium *Spirulina maxima* inhibited ERK1 activity and the generation of ERK1-induced ROS in response to LPS, resulting in decreased NF-κB expression as well as decreased NLRP3 activation as assayed by IL-1β cleavage [36]. Separately, in vitro and in a mouse model of atherosclerosis, miR-155 was implicated in promoting an ox-LDL-ERK1-NLRP3 signaling axis that worsened atherosclerotic inflammasome activation and worsened plaque formation—which could be selectively reversed with the ERK inhibitor U0126 [37]. Interestingly, in another inhibitor screening study, inhibitors of ERK1, but not ERK2, impeded rapid priming of NLRP3 in monocytes—which the authors reported to possibly be due not to phosphorylation, but instead to indirect ERK1-dependent regulation of NLRP3 ubiquitination levels [38]. In any regard, ERK1 in particular appears to be worthy of future study as a potential upstream pharmacological target for attenuation of NLRP3 inflammasome hyperactivation.

#### 2.1.2. Ubiquitination of NLRP3 and Regulatory Molecules Associated with Inflammasome Activation

The first post-translational modification to NLRP3 discovered was ubiquitination, and a wealth of subsequent studies have revealed NLRP3 ubiquitination controls both the stability of the NLRP3 protein and the priming process.

The C-terminal LRR domain has been shown to be subject to K63-linked polyubiquitination (performed by RNF125 amongst others), and the E3 ubiquitin ligase Cbl-b binds to these K63-ubiquitin chains and subsequently performs K48-linked ubiquitination of the NLRP3 K496 residue, leading to proteasomal degradation of NLRP3 and negatively regulating inflammasome assembly [39]. Similarly, FBXL2 was found to ubiquitinate the NLRP3 K689 residue, and LPS inhibits the interaction between FBXL2 and NLRP3 that leads to proteasomal degradation by this mechanism [40]. These two lysine residues represent the only two sites that have been functionally validated in regard to the effects of ubiquitination on NLRP3 stability and activation, but numerous other potential interaction partners in NLRP3 ubiqituination and deubiquitination as well as that of other components of the inflammasome have been reported. These are comprehensively reviewed in [41]. Of note, at least one report has shown JNK1-dependent phosphorylation of NLRP3-S194 plays a direct role in deubiquitination of NLRP3 and subsequent inflammasome activation, suggesting the potential for significant interplay between the phosphorylation and ubiquitination arms of NLRP3 post-translational modification that might be leveraged in development of targeted therapies [20].

### 2.2. Direct and Indirect Pharmacological Inhibition of NLRP3 Inflammasome Activation

As mentioned in the previous sections, targeting the molecular actors responsible for post-translational modification of NLRP3 to alter its persistence in response to inflammatory stimuli is likely to be a particular topic of interest in studies of NLRP3 regulation in the coming years. However, many agents have already been developed that show either direct activity against the NLRP3 protein or indirect activity (either on relevant signaling pathways or on other components of the NLRP3 inflammasome) and will continue to serve as valuable tools to glean insight into the attenuation of NLRP3 hyperactivation. A comprehensive list of these agents is elegantly reviewed in [42].

## 3. The NLRP3 Inflammasome and DNA Sensing

### 3.1. The Role of DNA in Autoimmune Pathology

A cornerstone of mounting a competent innate immune response relies on the ability of cytosolic and endosomal PRRs to identify RNA and DNA from foreign sources (particularly viruses, who often elude other immune counter-Fstrategies). Cytosolic DNA sensing has been the focus of recent exciting work, yielding insights into how established PRRs such as NLRP3, TLRs, and the retinoic-acid-inducible gene I (RIG-I)-like receptors can identify microbial nucleic acid sequences [43]. Cell-free DNA (cfDNA) has been studied in such diverse pathologies as atherosclerosis, primary Sjögren’s syndrome, rheumatoid arthritis, and systemic lupus erythematosus (SLE)—which are all characterized by: (1) persistent auto-inflammation driven by dysregulation; and (2) the presence of elevated cfDNA levels. These same features have been shown to be present in end-stage renal disease, as well as in the setting of dialysis [44,45]. Interestingly, homeostatic levels of cfDNA do not promote pathology, however it is hypothesized changes in methylation status, fragment length, and quantity contribute to the pathogenicity and immunogenicity of cfDNA. In solid organ transplantation, elevations in levels of cfDNA specifically derived from the donor allograft (donor-derived cell-free DNA, dd-cfDNA) is associated both directly with allograft injury, as well as serving as a risk stratification factor for poor clinical outcomes across solid organs in adult and pediatric recipients alike [46,47,48,49,50,51,52,53]. Perhaps most strikingly, in a multicenter cohort of prospectively recruited patients all of which presented with biopsy-confirmed T cell-mediated rejection of Banff class 1A or borderline rejection, dd-cfDNA scores above 0.5% at any time during surveillance were associated with significantly higher decline in estimated glomerular filtration rate (eGFR, a standard measure of renal allograft function), a nearly 20-fold increase in the rate of de novo donor-specific antibody (DSA) development, and a significant increase in the rate of rejection recurrence or worsening 3–6 months following dd-cfDNA measurement [53]. Numerous studies have now been published that reproducibly report elevated dd-cfDNA levels surrounding and during acute allograft rejection as well as the return of those levels to pre-episode baselines upon resolution of the rejection episode with treatment [51,54,55]. Based on these observations, dd-cfDNA may itself represent a danger signal that initiates an immune response in the form of dendritic cell (DC) activation. In a parallel vein, high levels of mitochondrial DNA (mtDNA) are found in the circulation of patients suffering from sepsis, trauma, and chronic organ-specific illnesses such as rheumatoid arthritis, gout, SLE, and hepatitis [56,57,58,59]. In transplantation, mtDNA is also released following allograft reperfusion and has also been shown to be a compelling danger signal that is recognized by the innate immune system, directly modifying the inflammatory response. mtDNA shares a lineage with prokaryotic DNA, and as such the similarities seen in transplantation between the effects of mtDNA and dd-cfDNA suggest that conserved immune response pathways might govern both. Of particular interest is the potential entry mechanism for dd-cfDNA into effector immune cells—one in vitro study has reported that only oxidized cfDNA fragments can enter cells, with non-oxidized fragments remaining in the extracellular space [60]. This implies that the manner in which dd-cfDNA is released or the degree of damage it sustains due to ROS as it enters the circulation may directly impact its ability to serve as an antigenic stimulus. To this end, this review turns its focus to a cell type repeatedly associated with localized accumulation of highly oxidized DNA in the circulation—the neutrophil.

### 3.2. Neutrophils and the NLRP3 Inflammasome—Casting a Wide NET

Neutrophils by proportion make up the majority of the leukocyte compartment. Neutrophil extracellular traps (NETs) are a major and fairly recent discovery in the pantheon of innate immune defense signals. NETs are formed when extracellular DNA is ejected from neutrophils and assembled alongside extracellular matrix proteins such as vimentin, histones, and ROS on the neutrophil surface –these proteins have the capacity to serve as self-antigens in autoimmune pathology as well as mediate a vast range of intracellular modifications and processes [61,62,63]. Pathologically, NETs promote thrombosis, play an active role in ischemia-reperfusion injury, and are directly involved in tissue fibrosis with aging [64,65,66,67]. In severe cases of COVID-19 NETs have been implicated as the main cause of vascular occlusion of the microcirculation of the kidneys, and it has been postulated that antagonism of the P2X14 purinergic receptor, which regulates ion flux, might provide an avenue for pharmacologic intervention in this regard [68,69]. Importantly, overabundance of NETs (NETosis) and dysregulation in NLRP3 inflammasome activation have both been associated with similar disease phenotypes—namely, atherosclerosis, ischemia-reperfusion injury and hypoxia-induced venous thromboembolism [70,71,72].

The underlying common denominator for NETosis appears to be peptidylarginine deiminase 4 (PAD4), which modifies histones to make them less able to bind DNA by replacing arginine residues with citrulline residues [73,74]. PAD4 loss-of-function has been demonstrated to protect against the above pathologies in mouse models [67,75]. In a recent and exemplary study, one group reports that PAD4 overexpression results in LPS-independent inflammasome activation upon nigericin (a potassium ionophore that acts as a potent NETosis effector) stimulation without affecting IL-1β, ASC or NLRP3 mRNA levels. These data suggest that the effect of PAD4 on the NLRP3 inflammasome is wholly post-transcriptional [76]. The authors conclude that the effect of PAD4 on increasing NLRP3 inflammasome protein is either due to a translation-dependent effect or the inhibition of the ubiquitin-mediated proteasomal clearance of NLRP3. In mouse models of NLRP3 deficiency, NET density and rate of formation is significantly impaired relative to wildtype—implying strongly that NLRP3 is a major component in NETosis. Finally, the authors draw compelling connections between NLRP3, NETosis, and the notion that NETs serve as vectors for promotion of acute and chronic autoimmune disorders. Previous work in type 2 diabetes implicates both elevation of neutrophil PAD4 and NETosis alongside increased NLRP3 inflammasome activation [77,78]. This group’s discovery of the formation of PAD4-dependent ASC speck structures that are expelled into NETs greatly advances the idea of NETs as reservoirs of self-antigenic activity in autoimmune disorders. ASC speck structures trapped in NETs have the potential for uptake by other cells and self-propagation of inflammasome assembly in those cells (particularly macrophages) [6,70,79].

More evidence for connections between NLRP3 inflammasomes, NETosis, and promotion of chronic systemic inflammation can be found in studies of diabetes. Neutrophils from diabetic patients have been shown to be overly prone to NETosis, and proteomic analysis of blood from diabetic patients with impaired wound healing and diabetic foot ulcers revealed a direct relationship between the level of NET components in the blood and wounds and the rate of infectious complication as well as severity of wound [77,80]. In diabetes, NLRP3 inflammasome hyperactivation in macrophages promotes both systemic inflammation and NETosis, which then leads to a feed-forward mechanism in which NETs produce elevated levels of ILs-1β and -18 and perpetuate an environment characterized by ever increasing inflammation, NETosis, and inflammasome activation [81]. A recent study comparing healthy individuals to diabetic patients with or without diabetic foot ulcers revealed that this cycle might be relieved or even broken by milk fat globule epidermal growth factor VIII (MFG-E8) [82]. This ubiquitous glycoprotein promotes increased phagocytosis of damaged neutrophils and other cells [83,84]. Exogenous MFG-E8 was previously observed to be associated both with decreased NLRP3 inflammasome activation and lower levels of IL-1β production through mediation of integrin β3 and P2X_7_ receptor interactions in renal and post-ischemic cerebral injury [85,86]. The authors further report that in a mouse model of diabetes, MFG-E8 promotes angiogenesis and wound healing—either through the demonstrated direct suppression of NLRP3 inflammasome activity and NETosis or by favorably altering the expression of the NLRP3-activating P2X7 purinergic receptor [82]. While more insight into the specific mechanism of action is needed, this study provides an excellent example of not only the connections between NLRP3, NETosis, and chronic inflammation in autoimmune disease—but also potential stratagems to counter these interrelated molecular pathologies.

## 4. NLRP3 Inflammasome Activation in End-Stage Organ Failure and Transplant

The interplay between chronic inflammation, NETs/NETosis, and NLRP3 inflammasomes leads directly to the idea that NLRP3 inflammasome hyperactivation is likely to underlie many of the end-stage disease conditions that result in the necessity for as well as post-transplantation complications related to alloimmune injury and rejection. In this section, this review aims to briefly highlight findings from solid organ-specific fields regarding NLRP3 inflammasome involvement in either pre- or post-transplant pathology. Literature on NLRP3 inflammasome activation in the specific fields of hematopoietic stem cell transplant [87,88,89], pancreatic islet cell transplant [90], and post-transplant complications such as graft-versus-host disease and the development of myeloproliferative neoplasms [87,91] is abundant, and as such re-direction to the superlative existing recent review treatments of these fields is necessary.

### 4.1. Liver

Hepatic ischemia-reperfusion injury (hIRI) is marked by precipitous increases in circulating factors that recruit neutrophils into the ischemic liver tissue, and represents a dire potential complication to both liver surgery and transplant procedures [92]. One study revealed that NLRP3, but not other components of the NLRP3 inflammasome, showed increased expression in hIRI tissue. In vivo, liver injury in response to 60 min of partial ischemia and subsequent reperfusion is significantly attenuated in animals lacking NLRP3, but not those deficient in ASC or caspase-1 [93]. Findings regarding tissue levels of IL-1β corroborated these observations, with only the loss of NLRP3 correlating with a decrease in IL-1β expression in hIRI. Interestingly, the authors report that this novel role for NLRP3 in promotion of pathologic hIRI in the liver appears to be independent of inflammasome assembly and not limited to bone marrow-derived cell populations. The authors instead implicate impaired neutrophil migration as a result of both deficient actin cytoskeleton assembly and Rac activation, and also acknowledge a potential role for NLRP3 in hepatic stellate cells in promotion of hIRI. Other studies in the field of hIRI come to somewhat different conclusions—potentially influenced by the relative degree of hIRI between protocols—and the understanding of the specific role of IL-1β in hIRI remains complicated by discrepant reports [94,95,96,97].

Findings in hepatic transplant also suggest prominent roles for NLRP3 itself as well as NLRP3 inflammasome activation and downstream signaling in hIRI-related pathology. One study identified monocyte expression of the purinergic P2X7 receptor in a cohort of liver transplant recipients undergoing controlled immunosuppression withdrawal, noting that P2X7 expression in inflammatory monocytes increased while the percentage of inflammatory monocytes remained constant in patients undergoing acute rejection [98]. Conversely, in patients that tolerated immunosuppression withdrawal, P2X7 expression remained stable. Extracellular ATP levels also followed this pattern, implicating both P2X7 and circulating ATP (both known to activate the NLRP3 inflammasome, as seen above in Section 3.2 in the discussion surrounding MFG-E8 in diabetes) in the acute phases of hepatic allograft rejection in response to unsuccessful withdrawal of immunosuppression. In a Bama minipig xenotransplantation model utilizing hypothermic machine-perfused donor after circulatory death livers, the combination of an addition of the direct NLRP3 antagonist MCC950 to both the perfusate and as 3 mg/kg intravenous regimen on days 2 and 3 post-transplant resulted in significantly decreased hIRI extent and superior function to control and MCC950 perfusate-only groups [99]. In a clinical liver transplant patient cohort, another group found NLRP3 expression to be strongly induced post-transplantation in hepatic allograft tissue [100]. Importantly, hyperexpression of intragraft NLRP3 was not only associated with clinical assessments of poor liver function, but also higher levels of neutrophil intravasation into the graft post-transplant. This observation is broadly consistent with the observations from the research studies detailed in Section 3.2 in other chronic autoimmune disease settings. This study went on to identify the telomere-independent repressor activator protein (RAP1) as a neutrophil-localized interaction partner for NLRP3 and the keratinocyte chemoattractant KC as a RAP1 interaction partner that was also elevated in intragraft expression post-transplant and was found to be an effector of early inflammation in acute allograft hIRI. NLRP3 inflammasome activation has also been definitively ascribed as a molecular cause of non-alcoholic fatty liver disease (NAFLD) to non-alcoholic steatohepatitis (NASH) progression—a major and rapidly increasing primary etiology for eventual liver transplant recipients. These findings are comprehensively reviewed in [101,102].

### 4.2. Lung

Lung transplant recipients face the most daunting 1- and 5-year survival rates among solid organ transplant recipients, largely due to high incidence of chronic rejection (also called chronic lung allograft dysfunction or CLAD, and previously widely referred to as bronchiolitis obliterans syndrome or BOS). A recent study shows N-myc-interactor expression is hyperactivated in CLAD patients and induces epithelial-to-mesenchymal EMT activation, leading to the characteristic pathologic fibrotic remodeling response [103]. A long history of literature in cardiac remodeling also implicates EMT and myofibroblast activation transduced by stress signaling through JNK and p38 MAPK pathways in deposition of fibrous tissue in response to myocardial insult—providing significant evidence for EMT and the myofibroblast response in promotion of solid organ pathologies like CLAD that are characterized by widespread fibrotic remodeling [35,104,105,106,107,108,109]. Myofibroblast activation has been shown to be dominantly controlled by TGF-β1/p38 MAPK signaling pathways, and as stated in Section 2.1.1 of this article TGF-β1/p38 MAPK signaling was also reported to specifically upregulate both TLR4 and NLRP3 expression in cardiac fibroblasts undergoing transition to the myofibroblast phenotype [35].

These observations were borne out by an exemplary 2013 study reported by the CTOT-03 investigators, who undertook a study of 23 patients with grade 3 primary graft dysfunction compared to frequency-matched controls (criteria: donor age and recipient primary disease diagnosis). Bronchoalveolar lavage fluid was obtained from both donors and recipients, and RNA isolated from the lavage fluid was analyzed by Affymetrix Human Gene 1.0 ST Array [110]. Eight total gene sets met the statistical criteria for significant upregulation, 7 of which were directly related to NLRP3 or the activation of the NLRP3 inflammasome (notable findings include TLRs, the IL1Rs, and the NF-kB signaling adaptor MyD88). Furthermore, when the group analyzed the relative frequency of individual transcripts, IL-1β and NLRP3 were the first- and second-most elevated in grade 3 PGD relative to controls—taken together, a resounding finding regarding the importance of the NLRP3 inflammasome in the mediation of lung allograft dysfunction and failure.

A recent study reveals that in a tracheal transplant mouse model of CLAD, intraperitoneal injection of 50 mg/kg/day of MCC950 inhibited NLRP3 inflammasome activation in the post-transplant setting [111]. This inhibition was accompanied by improved maintenance of tracheal luminal diameter and less fibrotic deposition in the orthotopic allograft, as well as a repolarization of the T cell profile away from pro-inflammation Th1/Th17 subclasses and toward the pro-tolerance Treg subclass. Addition of exogenous ILs-1β or -18 abolished MCC950-conferred protection and returned CLAD lesions to the level seen in vehicle controls, implicating NLRP3 activation in the progression of CLAD in allograft tissue. A second study in heterotopic tracheal transplantation in mice also confirms these findings, implicating the formyl peptide receptor 1 (FPR1, a G-protein-coupled receptor that fulfills pathogen- and inflammation-sensing functions) in transduction of inflammasome-activating signals that promoted CLAD [112]. Specifically, loss-of-function FPR1 mouse models showed impaired inflammasome activation due in part to decreased p38 MAPK/ERK signaling pathway activation as well as direct downregulation of TGF-β1 expression.

NLRP3 inflammasome activation in common pulmonary pathologies that lead to indication for transplantation has also been well-established. In a patient cohort comparing healthy control subjects to those with cystic fibrosis (CF) pre-transplant as well as CF patients after having undergone double lung transplantation, one group found that neutrophils from pre-transplant CF patients were metabolically distinct (more aerobic glycolysis) and displayed significantly higher levels of NLRP3 inflammasome activation relative to the other groups [113]. The group confirmed these observations in a murine CF model, noting that MCC950 inhibition of NLRP3 in vivo was associated with reduced lung IL-1β, reduced airway inflammation, and reduced duration of opportunistic *Pseudomonas* infection. A second study implicated both NLRP3 inflammasome activation and the immune response amplifier triggering receptor expressed on myeloid cells 1 (TREM-1) in modulating LPS-induced acute lung injury [114]. The group made use of the TREM-1-specific antagonist LR12 peptide to show significant improvements in post-LPS-induced acute lung injury survival, accompanied by decreases in NLRP3 inflammasome component expression and activation level as well as decreases in histologically assessed lung damage, oxidative stress, and immune cell intravasation into the lung tissue. This study identifies yet another potential upstream pharmacological target in TREM-1 that may prove fruitful in attenuating NLRP3 inflammasome activation in response to acute pulmonary insult.

### 4.3. Kidney

The role of NLRP3 inflammasome activation in renal ischemia/reperfusion injury (rIRI) and acute kidney injury that represents leading primary indications for renal transplantation has been well-established in recent years, and is beautifully reviewed in [115,116]. A wealth of studies have explored the role of NLRP3 inflammasome activation using a myriad of experimental models, nearly universally showing that inhibition of NLRP3 inflammasome activation significantly ameliorates disease progression and severity. A particularly strong example of this was an elegant study in a mouse model that conclusively showed: (1) NLRP3 inflammasome activation to be the primary factor underlying progressive renal failure caused by oxalate nephropathy; and (2) oxalate nephropathy-associated renal failure was due to NLRP3-mediated inflammation and not any effect on oxalate intestinal handling, metabolism rate, or general oxalate homeostasis [117]. Until fairly recently, however, the specific role of NLRP3 in human renal disease particularly in regard to renal transplantation remained unclear. In a 2016 retrospective cohort study, 1271 donor–recipient matched samples were undertaken to determine whether single nucleotide polymorphisms (SNPs) in NLRP3 were associated with renal allograft-related complications [118]. The group found that the gain-of-function rs35829419 SNP in donors was associated with a significantly elevated hazard ratio of 1.91 (95% CI 1.38–2.64, *p* < 0.001) for biopsy-proven acute rejection (BPAR) within the first-year post-transplant, while the loss-of-function rs6672995 SNP in recipients was associated with a significantly decreased hazard ratio of 0.73 (95% CI 0.55–0.97, *p* = 0.03) for BPAR within the first year post-transplant. No donor or recipient NLRP3 SNP was associated with any other evaluated clinical outcome in renal transplant. The results of this retrospective study make abundantly clear that targeting the activation of the NLRP3 inflammasome in renal transplant recipients is likely to be a crucial component in novel pharmacological strategies to combat allograft rejection. Speculation regarding the specific effects of NLRP3 inflammasome inhibition on the presentation of circulating biomarkers such as dd-cfDNA that signal allograft injury (and are particularly well-established in the routine clinical surveillance of renal transplant recipients) is enticing, and dd-cfDNA may well represent an ideal non-invasive and dynamic method for assessing the efficacy of such investigational therapies.

### 4.4. Heart

As previously referenced in Section 2.1.1 and Section 4.2, NLRP3 activation has been causally associated with TGF-β1/p38 MAPK signaling characteristic of pathologic cardiac remodeling (also reviewed in [119]). Roles for NLRP3 inhibitors in ameliorating the progression of pathologic cardiac remodeling (ischemic and non-ischemic) have been well-established in the literature and are comprehensively reviewed [120]. With regard to cardiac transplant, donation after brain death (DBD) remains the primary pool of available organs for allograft implantation, while donation after circulatory death (DCD) organs are increasingly being examined as viable clinical alternatives. One of the main differences between the two organ pools is that DCD heart tissues have significantly higher levels of NLRP3 expression. A proof-of-concept study in reperfused and reanimated mouse hearts reveals that DCD hearts had 80% higher tissue expression of NLRP3 than that seen in control beating-heart donor tissue [121]. Application of 50 μM 16673-34-0 (NLRP3 inhibitor) to the Langendorff perfusion reanimation apparatus significantly improved developed pressures and pressure gradients concomitantly with significant suppression of caspase-1 activity in the heart tissue. Interestingly, in a mouse model of myocardial infarction using loss-of-function strains for ASC and caspase-1, another group elegantly demonstrated that acute inflammation in response to myocardial ischemia/reperfusion injury (mcIRI) was due to inflammasome activation specifically in cardiac fibroblasts, but not in cardiomyocytes—underlying the idea that, at least in the heart, it is possible that the primary effectors of NLRP3-mediated myocardial injury are not circulating nor resident immune cells, but are instead tissue-resident cardiac fibroblasts that carry out immune-related functions [35]. Whether these observations eventually hold true in other organs as well is a potentially rich matter for further study, and may yield significant advances in our understanding of the relative impact of NLRP3 inflammasome activation in non-traditional cell types—particularly given the ready availability of single-cell RNA sequencing workflows that are so effective at identifying relatively rare cellular populations.

### 4.5. Skin

Pathologic autoimmune responses to dermal allografts were also found to have an NLRP3 inflammasome dependence in one study in a mouse model of allogeneic skin transplantation [98]. In this model, the host’s antigen-presenting cells demonstrated the ability to alter circulating ATP levels in a non-cell death-dependent manner that relies on the P2X7 purinergic receptor. ATP serves to initiate a feedback mechanism on the P2X7 in this model that leads to downstream activation of the NLRP3 inflammasome and the production, processing, and secretion of IL-18—with the ultimate consequence of promotion of the Th1/IFN-γ alloimmune response that results in graft injury. The authors likewise note that ablation of IL-18 results in a repolarization of the graft-infiltrating T cell population away from CD8 subpopulations and toward tolerance-promoting Treg subpopulations. This study again suggests strongly that modulation of P2X family receptor signaling might be a particularly effective method to reduce NLRP3 inflammasome activation that leads to the signaling cascades that govern solid organ allograft rejection, and that the P2X7-NLRP3 activation pathway in particular is conserved in other non-transplant-related autoimmune disorders.

## 5. Summary and Future Directions

This review of the NLRP3 inflammasome has attempted to cover some broad ground—ranging from structure and function of the NLRP3 inflammasome itself through its connections to cell-free DNA sensing and into observations in solid organ transplantation that are intended to provide a holistic overview of the understanding of NLRP3 in this very specific field. NLRP3’s ability to recognize and mount a competent innate immune response against a wide variety of toxins, flagellin, and peptidoglycan, aberrant proteins formed by cfDNA, and cfDNA/mtDNA itself signifies that the mechanistic potential for intervention in NLRP3 inflammasome-mediated signaling processes that lead to solid organ allograft pathology and negative clinical outcomes is quite vast indeed. Improved understanding of how NLRP3 inflammasome activation is spatially and temporally regulated during the acute and chronic post-transplant period in the clinic is of paramount importance to the field, and non-invasive biomarker surveillance in the form of donor-derived cell-free DNA is uniquely positioned to support this aim. With high-quality, prospective, multicenter clinical studies either published or well underway in every solid organ transplant patient population, dd-cfDNA’s obvious connections to NLRP3 inflammasome activation and regulation poise this technology to move even further to the forefront of technologies enabling superior allograft surveillance and patient outcomes.

## Data Availability

Not applicable.

## Abbreviations

ASC, inflammasome adaptor protein apoptosis-associated speck-like protein containing CARD; ATP, adenosine triphosphate; FADD, FAS-associated death domain protein; HMGB1, high-mobility group box 1; JAK/STAT, Janus kinase/signal transducers and activators of transcription; IFN, interferon; IFNAR, interferon-α/β receptor; IL, interleukin; MALT1, mucosa-associated lymphoid tissue lymphoma translocation protein 1; MAMP, microbe-associated molecular pattern molecule; MLKL, mixed-lineage kinase domain-like protein; MyD88, adaptor molecules myeloid differentiation primary response 88; Nek7, NIMA-related kinase 7; NF-κB, nuclear factor-κB; NLRP3, nucleotide-binding oligomerization domain, leucine-rich repeat and pyrin domain-containing protein 3; P2X, purinergic receptor 7; PAMP, pathogens-associated molecular pattern; RIP1, receptor-interacting protein 1; ROS, reactive oxygen species; TLRs, Toll-like receptors; TNFR, tumor necrosis factor receptor; TRIF, toll/IL-1 receptor homology (TIR)-domain-containing adapter-inducing interferon-β; TXNIP, thioredoxin-interacting protein.
